# Development of a Deep Learning Model for Hip Arthroplasty Templating Using Anteroposterior Hip Radiograph

**DOI:** 10.3390/jcm14248689

**Published:** 2025-12-08

**Authors:** Siwadol Wongsak, Tanapol Janyawongchot, Nithid Sri-Utenchai, Dhammathat Owasirikul, Suphaneewan Jaovisidha, Patarawan Woratanarat, Paphon Sa-Ngasoongsong

**Affiliations:** 1 Department of Orthopedics, Faculty of Medicine Ramathibodi Hospital, Mahidol University, Bangkok 10400, Thailand; siwadolrama@hotmail.com (S.W.); jan.tanapol@gmail.com (T.J.); pataraw@yahoo.com (P.W.); 2 Department of Orthopaedics, Sisaket Hospital, Sisaket 33000, Thailand; 3 Chakri Naruebodindra Medical Institute, Faculty of Medicine Ramathibodi Hospital, Mahidol University, Samut Prakan 10540, Thailand; nithid.sri@mahidol.ac.th; 4 Department of Radiological Technology, Faculty of Medical Technology, Mahidol University, Nakorn Pathom 73170, Thailand; dhammathat.owa@gmail.com; 5 Department of Diagnostic and Therapeutic Radiology, Faculty of Medicine Ramathibodi Hospital, Mahidol University, Bangkok 10400, Thailand; rasjv@yahoo.com

**Keywords:** artificial intelligence, machine learning, preoperative templating, plain radiograph, on-screen templating

## Abstract

**Background:** Preoperative templating is an essential step in hip arthroplasty (HA), guiding implant selection and reducing surgical complications. It is typically performed using acetate templates or digital software. These methods, however, depend on the surgeon’s experience and may be limited by cost and availability. This study aimed to develop and validate a deep learning (DL) model using plain radiographs to predict implant sizes in HA. **Methods:** This retrospective study included patients who underwent primary HA using a cementless CORAIL^®^ femoral stem and PINNACLE^®^ acetabular cup. The DL model was trained on 688 preoperative anteroposterior (AP) hip radiographs and validated temporally on 98 additional cases. Implant sizes predicted by the DL model were compared with on-screen templating (acetate templates overlaid on digital images). The actual implanted size was used as the reference standard. Accuracy, mean absolute error (MAE), and root mean square error (RMSE) were calculated. Logistic regression was performed to identify factors influencing prediction accuracy. **Results:** The DL model showed higher accuracy than the on-screen templating for the acetabular cup (88.9% [77.4% to 95.8%] vs. 83.3% [70.7% to 90.2%]) and femoral stem components (85.7% [77.2% to 92.0%] vs. 81.6% [72.5% to 88.7%]), while the on-screen method performed better for the bipolar head (93.2% [81.3% to 98.6%] vs. 72.7% [57.2% to 85.0%]). MAE and RMSE were comparable between the methods for acetabular and femoral stem components (all *p* > 0.05), with statistically significant differences observed only in the bipolar head (*p* < 0.01 and 0.02, respectively). Although logistic regression analysis showed trends toward higher accuracy in acetabular size prediction among women and those with shorter height, no demographic factors were statistically significant predictors of accuracy. **Conclusions:** A DL model using only plain radiographs can accurately predict implant sizes in HA, particularly for the acetabulum and femoral stem. These findings suggest that the DL-based model could be a useful tool in preoperative planning. With further refinement to improve generalizability, this approach could be useful in a routine clinical setting in the future.

## 1. Introduction

Preoperative templating in hip arthroplasty (HA) is an essential step in surgical planning for HA, guiding appropriate implant size selection and joint biomechanics restoration. Accurate templating improves component stability and positioning, minimizes leg length discrepancy, and reduces complications, which enhance long-term clinical outcomes [1,2,3]. Moreover, accurate preoperative sizing can improve surgical efficiency, minimize costs, and reduce operative time [4].

Templating methods have evolved from traditional acetate template overlays on printed radiographs to digital templating systems. However, acetate templating using plain radiographs often lacks precision due to variations in radiographic magnification [5]. Although digital templating has demonstrated good reliability and validity [6,7,8], it requires specialized and expensive software available across workstations, and regular software updates may be needed. Generally, a hybrid approach, such as using acetate templates on calibrated digital radiographs (on-screen templating method), provides reliable results and can be used as a practical alternative to digital platforms in resource-limited settings [9,10].

Recent advances in artificial intelligence (AI), especially deep learning (DL), have enabled automated interpretation of medical images commonly used in orthopedics, such as radiographs, computed tomographic scans, and magnetic resonance imaging scans [11]. DL offers an automated, user-independent approach that reduces reliance on surgeon experience and minimizes errors associated with manual templating, such as inaccuracies from magnification adjustments and variability in digital template applications. AI models trained on large datasets have shown high accuracy in identifying implant types and supporting preoperative planning [12,13]. Previous studies also demonstrated AI’s potential ability to determine dislocation risk and implant loosening in total hip arthroplasty (THA) [14,15]. In recent studies, AI-assisted three-dimensional planning has outperformed traditional methods in implant size prediction, offering greater reliability compared to conventional two-dimensional templating [16,17,18]. Despite these promising results, current AI approaches face several limitations. Many models rely on high-quality imaging, such as computed tomography (CT), and their performance may be reduced in patients with complex anatomy, such as developmental dysplasia of the hip (DDH) [19], without statistical differences in long-term functional outcomes compared to the traditional method [20]. To the best of our knowledge, only Kim et al. demonstrated the efficacy of DL application for automated cementless THA planning with hip anteroposterior (AP) radiographs [21].

This study aimed to develop and validate a DL model for predicting implant sizes in hip arthroplasty using plain radiographs. We hypothesized that the DL model could predict implant size with accuracy comparable to the standard templating method. Therefore, we aimed to (1) access and compare predictive accuracy of the DL model and the standard on-screen templating method and (2) identify patient-related factors associated with prediction accuracy.

## 2. Materials and Methods

This retrospective study was conducted at a medical university hospital. Prior approval was obtained from the Institutional Review Board (IRB No. COA.MURA2022/175 and COA.MURA2025/178) in accordance with the ethical principles outlined in the Declaration of Helsinki.

*Patient Cohort and Eligibility Criteria.* Patients who underwent primary hip arthroplasty between 1 January 2017 and 28 February 2022 (phase 1 study) and between 8 March 2022 and 7 March 2025 (phase 2 study) were recruited. Only cases performed with a CORAIL^®^ cementless femoral stem and a PINNACLE^®^ acetabular cup (DePuy Synthes, Warsaw, IN, USA) were included to standardize implant design. All operations were conducted using the anterolateral approach in the lateral decubitus position. Intraoperative implant size selection was based on implant stability and confirmed using fluoroscopic guidance. The inclusion criteria were (1) availability of preoperative anteroposterior (AP) hip radiographs and (2) complete operative documentation, including implant sizes. The exclusion criteria included (1) missing or incomplete radiographic or operative data; (2) inadequate radiograph for analysis, such as excessive femoral rotation, underexposure, or motion artifact; and (3) intraoperative complications requiring deviation from standard procedures, such as periprosthetic fracture with adjunctive cerclage wiring.

*Radiographic acquisition and preprocessing.* One of the authors manually screened the radiographic datasets to ensure adequacy for DL input, including anatomical completeness and consistent positioning. In our institution, well-trained technicians obtained the preoperative AP hip radiographs using the same radiographic machine in the orthopedic outpatient clinic and the same radiographic setup protocol. The patients were placed in the supine position with 15-degree internal rotation of both legs to optimize femoral neck visualization, without calibration markers. The X-ray tube was set with a standardized source-to-image receptor distance (SID) of 110 cm. The beam was centered at the midpoint between the symphysis pubis and the line connecting both anterosuperior iliac spines. The source-to-object (as a hip joint) distance (SOD) depended on the patients’ body size, varying from 90 to 95 cm. Therefore, the magnification factor was calculated by dividing SID by SOD, resulting in a magnification factor between 1.15 and 1.22.

**Figure 1:**
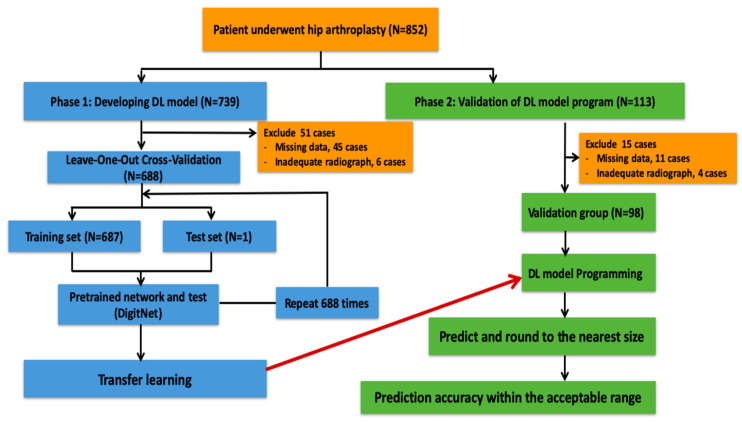
The study workflow. The DL model was developed in phase 1 using data from 688 radiographs and the leave-one-out cross-validation method. The final model from phase 1 was then validated in phase 2 with another 98 radiographs.

*Study Workflow and Dataset Division.* Figure 1 shows the study workflow. This study was conducted in two phases: model development and external validation.

*Phase 1—Development of the DL Model.* Of the 739 preoperative hip radiographs recruited (during the period from 1 January 2017 to 28 February 2022), 51 were excluded due to missing data (*n* = 45) and inadequate quality of radiographs (*n* = 6). Therefore, a total of 688 preoperative hip radiographs were used to train the DL model based on the Diginet convolutional neural network (CNN) architecture, a DL CNN that can learn and recognize common features, such as natural images of digits [22] (Figure 1 and Table 1). The model was implemented and trained using MATLAB R2023a (MathWorks Inc., Natick, MA, USA). Each radiograph was standardized by manual cropping to isolate the hip area, resizing to 24 × 24 pixels, converting to grayscale, and normalizing to a 0–1 intensity scale. The selected hip area was generally chosen from the side with hip disease. However, in the femoral neck fracture, the hip area was selected from the contralateral hip joint due to the anatomical change from fracture displacement. The implant size, as documented in the operative records, served as the ground truth. Separate CNN models were developed to predict the implant sizes of the acetabular cup, bipolar head, and femoral stem. A regression-based approach was applied to train the model to learn the continuous ordinal relationship between implant sizes (Supplementary Table S1) and address the imbalanced dataset problem caused by the rarity of very small and very large prosthesis sizes in the normal distribution of the general population’s size. For each model, the output layer consisted of a single neuron with a linear activation function, enabling continuous prediction of implant sizes. The models were trained using the root mean square error (RMSE) loss function, and optimization was performed using the Adam optimizer with a fixed learning rate of 0.001. Hyperparameter tuning was performed by training each model under various combinations of optimization parameters (Supplementary Table S1). Leave-one-out cross-validation (LOOCV) was employed to maximize data utilization. RMSE was used as the sole optimization criterion for DL model development because the prediction targets were on an ordinal-to-continuous scale, where the magnitude of error was clinically meaningful. RMSE directly reflects the deviation between the predicted and reference values, and it also penalizes large errors with the quadratic penalty, which is consistent with clinical risk considerations. For each configuration, the model achieving the lowest average RMSE across all LOOCV folds was selected as the final model for each implant component.

*Phase 2—Validation and Comparison with the on-screen Templating method.* Of the 113 preoperative radiographs recruited during a later non-overlapping period (8 March 2022 and 7 March 2025) at the same institution, 15 were excluded due to missing data (*n* = 11) and inadequate radiographic quality (*n* = 4) (Figure 1). Therefore, a total of 98 preoperative radiographs, representing a temporal validation cohort, were used for validation following the model training and optimization. All radiographs were obtained with the same digital radiography system and identical acquisition parameters. Its predictive performance was compared with an on-screen templating method, in which acetate templates were manually overlaid onto digitally displayed radiographs on LCD monitors. A standard 20% oversized acetate template set of the Corail^®^ total hip system and the Pinnacle™ acetabular cup system (DePuy, Johnson & Johnson, Warsaw, IN, USA) was placed over the digital hip images on the computer screen (on-screen) provided by Picture Archiving and Communication System (Synapse PACS, version 7.4). One of the authors performed digital image scaling using digital calibration within Synapse PACS software to perfectly match the ruler in the digital images and the ruler in the acetate templates [23,24]. On-screen templating was performed by a single orthopedic surgeon with more than 10 years of experience in hip arthroplasty on two separate occasions, spaced four weeks apart, to evaluate intra-observer reliability (Table 2). The surgeon was blinded to the operative record and prior templating results. Model performance was evaluated using “exact match” (same predicted and implanted size) and “accurate prediction” (within ±1 size step for femoral stem, equivalent to ±2 mm for acetabular and bipolar components). The implanted size, as recorded in the operative note, was used as the reference standard. The required validation sample size was estimated using a single-proportion formula based on previously reported accuracy by Shin et al. [10], as the accuracy within ±1 size was 96.6% for the cup size and 97.8% for the stem size. Using a margin of error and confidence interval of 5% and 95%, the required minimum sample size in this cohort was estimated at 51 and 34 cases, respectively.

*Data Analyses.* All statistical analyses were performed using STATA software (version 17.0; StataCorp LLC, College Station, TX, USA). Intra-observer reliability was assessed by calculating the intraclass correlation coefficient (ICC) using a two-way random-effects model with absolute agreement (ICC (2,1) criteria). Model prediction performance was evaluated using mean absolute error (MAE) and root mean square error (RMSE), as these metrics appropriately capture the magnitude of and variability in prediction errors for evenly spaced class intervals. In phase 2, the accuracy of the DL model and on-screen templating method were presented as the number of cases with corrected prediction and percentage with 95% confidence interval (CI) and were compared with MAE and RMSE. Bland–Altman plot was used to assess the agreement between the methods. Logistic regression analysis was performed as an inferential method to evaluate associations between demographic variables (gender, weight, height, and BMI) and the accurate prediction of the DL model in the validation phase. Statistical significance was defined as *p* < 0.05.

## 3. Results

### 3.1. DL Model Performance

**Figure 2:**
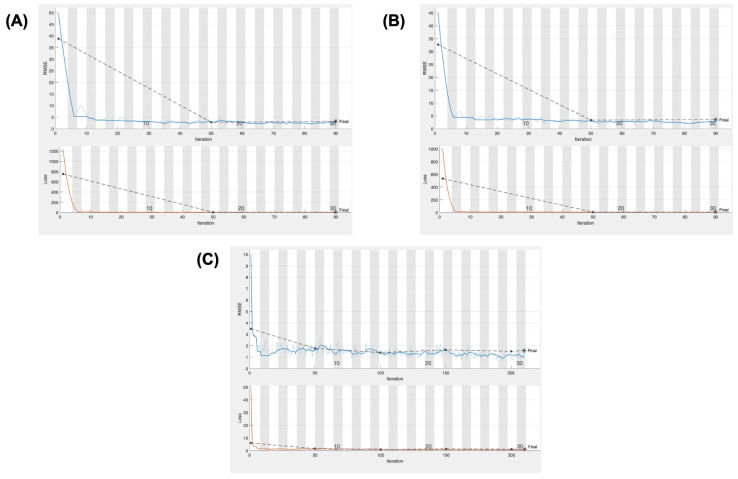
Training curves showing RMSE (**top**) and loss (**bottom**) across iterations for (**A**) acetabular cup, (**B**) bipolar head, and (**C**) femoral stem prediction models.

RMSE and loss values across iterations for each implant component were tracked during the training process. After hyperparameter tuning, the optimal CNN architecture was selected for each implant component based on the lowest RMSE. All models showed a steep drop in error during the early stages of training, followed by a stable plateau, suggesting that the model learned efficiently and converged. The final model achieved an RMSE value of 1.35 ± 0.04 (mean ± standard deviation) for the femoral stem. For the acetabular cup and bipolar head, the RMSE values were 2.20 ± 0.08 mm and 2.00 ± 0.09 mm, respectively. These values suggest that the model could estimate implant sizes with reasonable consistency before validation on the external dataset. (Figure 2).

### 3.2. Validation Phase

A total of 98 preoperative hip radiographs from 98 patients were included. The mean age was 70.1 years; 75 patients (76.5%) were women and 23 patients (23.5%) were men. The mean height and weight were 155.4 cm and 58.0 kg, respectively, with a mean BMI of 23.9 kg/m^2^. The most common preoperative diagnosis was femoral neck fracture in 46 patients (46.9%), followed by osteonecrosis (ON) in 32 patients (32.7%), osteoarthritis (OA) in 11 patients (11.2%), and DDH in 9 patients (9.2%). There was no significant difference in demographic characteristics between the development and temporal validation cohorts (all *p* > 0.05) (Table 1). The implanted component size distributions in the development and validation cohorts are summarized in Supplementary Table S2.

The on-screen templating method demonstrated excellent intra-observer reliability. The value for acetabular size prediction was 0.90 (95% confidence interval (CI): 0.83–0.94), the value for bipolar head size was 0.99 (95% CI: 0.99–1.00), and the value for femoral stem size was 0.95 (95% CI: 0.93–0.97).

**Figure 3:**
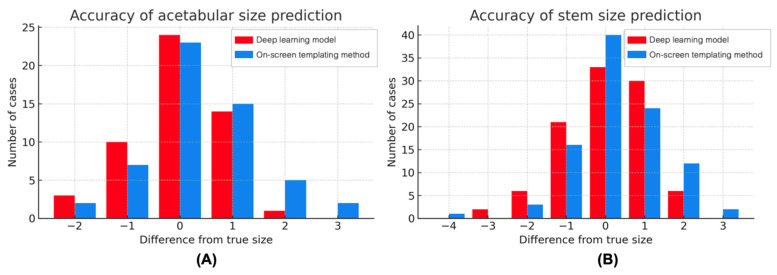
Error distribution for acetabular size (**A**) and stem size (**B**) prediction between the DL model and the on-screen method.

When comparing accuracy against the true implanted size, the DL model demonstrated comparable or higher performance than the on-screen templating method in predicting acetabular and stem sizes (Figure 3). For the acetabular component, the DL model achieved an exact match in 44.4% of cases and an accurate prediction in 88.9% of cases, slightly outperforming the on-screen method (42.6% exact match; 83.3% accurate prediction). For the femoral stem, both methods achieved the exact match rate (33.8% vs. 40.8%), while the DL model showed higher accurate prediction (85.7% vs. 81.6%). In contrast, for bipolar head prediction, the on-screen method outperformed the DL model in both exact match (61.4% vs. 43.2%) and accurate prediction (93.2% vs. 72.7%) (Table 3).

The error distributions for each implant component are summarized in Supplementary Table S3. Both the DL model and the on-screen method achieved most predictions within ±1 size. The DL model showed a slight undersized trend for the acetabular component and greater variability for the bipolar head, whereas the on-screen method produced more centered predictions. For the femoral stem, both methods demonstrated symmetrical error patterns without directional bias. All bipolar head cases were performed for femoral neck fracture without chronic hip osteoarthritis. The model achieved 70.0% accuracy for heads ≤ 42 mm, 78.3% for heads 43–45 mm, and 63.6% for heads ≥ 46 mm (Supplementary Table S4). Accuracy was highest in the mid-size range, likely reflecting better representation of these sizes in the training dataset.

**Figure 4:**
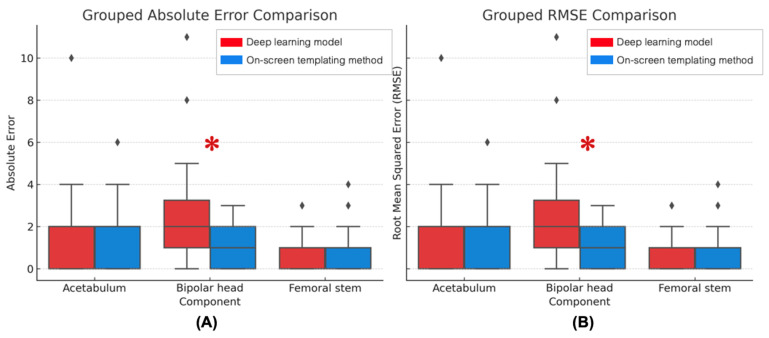
Comparison between the DL model and the on-screen method for each component. (**A**) Mean Absolute Error and (**B**) Root Mean Square Error (RMSE). Bipolar head component showed significant lower error in on-screen method compared to DL model (* statistically significant difference at *p* < 0.05).

**Figure 5:**
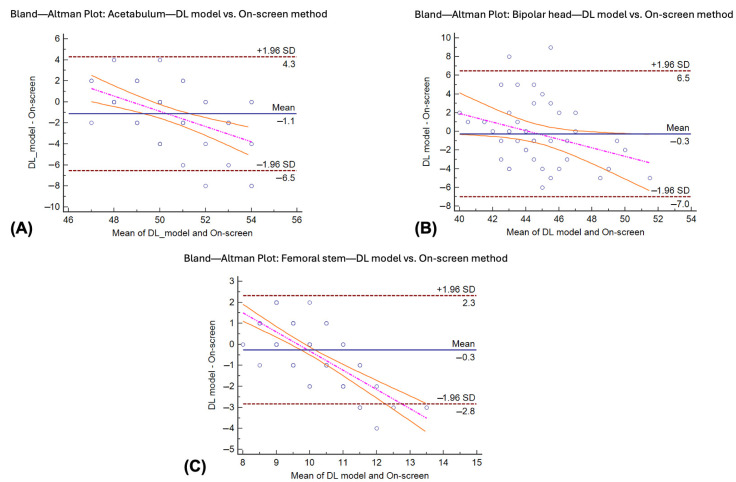
Bland–Altman plots between the DL model and the reference standard (on-screen templating method) for the (**A**) acetabular cup, (**B**) bipolar head, and (**C**) femoral stem. The pinked dotted and orange curved line in each figure showed the regression analysis for proportion bias (regression line).

MAE and RMSE were used to assess the prediction precision for each method. For the acetabular component, both the on-screen and DL models yielded the same MAE (1.56 mm), although the DL model showed slightly higher RMSE (2.58 vs. 2.24 mm), suggesting greater variability in its predictions. For the bipolar head size, the on-screen method outperformed the DL model, with lower MAE (1.18 vs. 2.25 mm) and RMSE (1.49 vs. 3.12 mm). For the femoral stem, the results were comparable between the methods. The DL model showed nearly identical MAE (0.83 vs. 0.82 size) and slightly lower RMSE (1.09 vs. 1.17 size), implying slightly better consistency (Table 4). Paired *t*-tests indicated no statistically significant differences between methods for acetabular (*p* = 1.00) and femoral stem predictions (*p* = 0.93). However, the bipolar head component showed significantly lower errors with the on-screen method (*p* < 0.01), reflecting greater precision and reliability of the on-screen method (Figure 4). Figure 5 shows the Bland–Altman plot between the results from the DL model and the on-screen method in all three components of prediction. All DL models demonstrated a linear decreasing pattern with the standard on-screen method, as DL models had a trend to predict a larger size in small implants and predict a smaller size in large implants. However, all DL models revealed the best agreement with the on-screen method in the mid-sized implant.

The univariate logistic regression revealed female gender (odds ratio (OR) = 7.60, *p* = 0.03) and lower height (OR = 0.88, *p* = 0.03) as significant predictors of acetabular component prediction accuracy. However, in the multivariate analysis, which included both variables, neither remained statistically significant (OR = 3.03, *p* = 0.35 and OR = 0.91, *p* = 0.21, respectively). Body weight and BMI were not significantly associated with prediction accuracy. For femoral stem accuracy, the univariate analysis did not show statistical significance among the predictors (Table 5 and Table 6).

## 4. Discussion

This study demonstrates that a DL model developed from a hip AP radiograph can achieve comparable accuracy to the conventional on-screen templating method in predicting implant sizes for hip arthroplasty from radiographs, particularly for the acetabular and femoral stem components. The DL model showed higher exact match and accurate prediction rates for these components, while the on-screen method performed better in predicting the bipolar head size.

To evaluate our DL model’s performance in practice, we compared the RMSE values between the internal and temporal holdout validation datasets. For the acetabular component, the RMSE increased slightly in the temporal holdout set, which suggests that the model remains reasonably accurate but may be slightly less precise in real-world use. However, the model’s performance in predicting femoral stem size was more stable in both the internal and temporal holdout datasets. The decreasing RMSE value in the temporal holdout group suggests that the model may generalize well for this component, which may be caused by clearer anatomical features and less variation in proximal femoral morphology. Similar observations have been noted in other machine learning (ML) studies, where model performance tends to decline when tested on external datasets, likely due to differences in patient characteristics or data acquisition that the model was not exposed to during training [25,26]. Moreover, a technical factor that may explain the RMSE differences is how the predictions were handled. During DL model development, the model produced continuous values from the regression framework, e.g., size 8.23, while in the temporal validation phase, the predictions were rounded to the nearest implant size, e.g., size 8.23 to size 8. This rounding can introduce an error that may affect RMSE and widen variability.

Regarding the agreement between the DL model and the standard on-screen method, the results from this study showed that there was a linear decreasing pattern relationship between all DL models and the on-screen method with the best agreement in the averaged implant size (Figure 5). These findings indicated that the DL model’s error consistently increased with extremely small and large implant sizes. This model error could also be explained by the small sample size of these uncommon sizes in this study.

Our performance was also comparable to that reported by Kim et al. [21], who demonstrated the accuracy of a DL CNN model using hip AP radiographs for predicting acetabular and femoral components within one size as 78.9% and 70.9%, respectively. This comparable outcome signified the benefits of radiographic image-based DL models in THA templating compared to the conventional on-screen templating method in general clinical practice. Ding et al. [7] reported exceptionally high accuracy in preoperative planning using AI-HIP software (Beijing Changmugu Medical Technology Co., Ltd., Beijing China), with 94.0% agreement for acetabular component size and 87.7% for femoral stem size, significantly outperforming traditional 2D manual templating (65.2 and 58.9%, respectively). These findings highlight the benefits of CT-based planning in capturing complex anatomical details, especially in patients with abnormal bone morphology. However, the practical use of the systems is limited by the requirement for additional imaging (CT), which may not be routinely obtained in daily practice. In contrast, our study demonstrates that the DL model can achieve comparable accuracy using only plain radiographs, with 88.9 and 85.7% accuracy in predicting acetabular and femoral stem sizes, respectively. This positions our model as a more practical solution for real-world applications, particularly in settings where access to CT imaging or specialized planning platforms is limited. Our method requires no additional imaging beyond routine AP radiographs, making it faster, more cost-effective, and easier to integrate into daily clinical workflows with reduced radiation exposure to patients. These advantages emphasize the potential of radiograph-based DL models as a practical alternative to high-resource 3D AI systems, especially in high-volume or resource-limited settings.

However, the results of this study revealed that the DL model’s accuracy in predicting the bipolar head size, measured by MAE and RMSE, was significantly lower than that of the standard on-screen templating method (Table 4, *p* < 0.05). This can be explained by several factors. First, the sample size for bipolar head validation was too small (*n* = 44). Second, the DL model in this study was developed from radiographs without calibration markers, resulting in radiographic magnification errors in measuring the true size of the femoral head. Moreover, the lower predictive performance of our DL model could be related to the impact of low-resolution images as plain radiographs in this study. Therefore, a larger dataset with calibrated radiographs with improved quality is needed to strengthen the predictive performance of a future DL model.

Regarding the logistic regression analysis for the predictors associated with accurate reduction in the validation cohort (phase 2) (Table 5 and Table 6), our results did not show any statistically significant differences with the multivariate analysis. These findings were not aligned with those of Zampogna et al. [27], who demonstrated a better accuracy of a supervised machine learning model with demographic and clinical variables. This can be explained by the ability of the DL CNN model in this study. Although there was a significant difference in pelvic anatomy between genders [28], DL models often demonstrate superior performance compared to traditional supervised machine learning models in the analysis of orthopedic images because they can automatically extract complex features from raw data [29]. These findings highlight the value of the DL application in orthopedics [11].

This study has several limitations. First, the model was developed and validated from a single-center dataset with a single acquisition protocol. Moreover, performance was evaluated against on-screen templating methods using implant sizes within one system, with accuracy for the bipolar head remaining lower. Therefore, this may affect its generalizability across other populations or imaging protocols. Second, although the preoperative radiographs in this study were obtained using a standard radiographic protocol, calibration markers were not used to standardize magnification, which may affect the performance of the DL model. Lastly, our DL model did not verify long-term postoperative clinical outcomes, such as implant loosening and functional outcomes. Therefore, further external validation and multi-center testing, following standard ethical considerations and data security for the clinical use of AI systems, with long-term follow-up data and clinical variable integration, are needed to confirm and improve the model’s performance [30]. Despite this limitation, the DL model still performed well compared to the standard on-screen templating method. This suggests that the model could handle image variability, which is a practical advantage in clinical use where perfect standardization is not always achievable.

## 5. Conclusions

This study demonstrates that a DL model trained on standard AP radiographs can predict implant sizes in hip arthroplasty with high accuracy comparable to conventional on-screen templating methods. Without the need for CT imaging, manual templating, or additional software, the model offers a streamlined and objective approach to preoperative planning.

While further development and validation are still needed, this radiograph-based AI approach shows promise as a practical tool in future clinical workflows. With continued refinement, such as incorporating multi-view imaging, larger datasets, or integration with clinical decision systems, this model could support more consistent, efficient, and accessible templating across a variety of healthcare settings. Therefore, we recommend using this DL model in clinical settings for THA due to its cost-effectiveness and reduced time-consuming tasks for preoperative planning.

## Figures and Tables

**Table 1:** Demographic data of the development (*n* = 688) and validation (*n* = 98) cohorts.

Parameters	Development Cohort (*n* = 688)	Validation Cohort (*n* = 98)	*p*-Value
Age (year) *	69.3 (14.8)	70.1 (13.9)	0.58
Gender **			0.94
Male	168 (24.4%)	23 (23.5%)	
Female	520 (75.6%)	75 (76.5%)	
Height (cm.) *	156.9 (8.3)	155.4 (8.1)	0.11
Weight (kg.) *	57.7 (12.4)	58.0 (12.6)	0.81
BMI (kg/m^2^) *	23.4 (4.4)	23.9 (4.4)	0.33
Diagnosis **			0.22
Osteoarthritis	109 (15.9%)	11 (11.2%)	
Osteonecrosis	175 (25.4%)	32 (32.7%)	
DDH	44 (6.4%)	9 (9.2%)	
Femoral neck fracture	360 (52.3%)	46 (46.9%)	

* Continuous data presented as mean (S.D.). ** Categorical data presented as N (%).

**Table 2:** Intra-observer agreement for size prediction using the on-screen templating method on 4-week interval measurements.

	Component		
	Acetabulum	Bipolar Head	Femoral Stem
ICC	0.90 (0.83–0.94)	0.99 (0.99–1.00)	0.95 (0.93–0.97)

Value presented as ICC (95% confidence interval).

**Table 3:** Accuracy for implant size prediction.

	Acetabulum (*n* = 54)		Bipolar Head (*n* = 44)		Femoral Stem (*n* = 98)	
	DL Model	On-Screen	DL Model	On-Screen	DL Model	On-Screen
Actual size	24/44.4% (30.9% to 58.6%)	23/42.6% (29.2% to 56.8%)	19/43.2% (28.4% to 59.0%)	27/61.4% (45.5% to 75.6%)	33/33.7% (24.4% to 43.9%)	40/40.8% (40.0% to 51.2%)
Accurate prediction	48/88.9% (77.4% to 95.8%)	45/83.3% (70.7% to 90.2%)	32/72.7% (57.2% to 85.0%)	41/93.2% (81.3% to 98.6%)	84/85.7% (77.2% to 92.0%)	80/81.6% (72.5% to 88.7%)

Value presented as number of cases with correct prediction/accuracy (95% confidence interval).

**Table 4:** MAE and RMSE comparison between the DL model and the on-screen method.

Component	Mean Absolute Error (MAE)			Root Mean Square Error (RMSE)		
	DL Model	On-Screen Method	*p*-Value	DL Model	On-Screen Method	*p*-Value
Acetabulum	1.56 (1.04–2.15)	1.56 (1.11–2.00)	1.00	2.58 (1.59–3.51)	2.24 (1.76–2.69)	0.57
Bipolar head	2.25 (1.66–2.93)	1.18 (0.91–1.45)	<0.01 *	3.12 (2.18–4.06)	1.49 (1.24–1.73)	0.02 *
Femoral stem	0.83 (0.68–0.97)	0.82 (0.65–0.99)	0.93	1.09 (0.94–1.24)	1.17 (0.98–1.36)	0.51

Values presented as means (95% confidence intervals). * significant difference at *p* < 0.05.

**Table 5:** Univariate and multivariate logistic regression analysis for prediction accuracy of acetabulum size (*n* = 54).

Predictor	Univariate Analysis			Multivariate Analysis		
	OR	95% CI	*p*-Value	OR	95% CI	*p*-Value
Female gender	7.6	1.21–47.60	0.03 *	3.03	0.30–30.26	0.35
Height	0.88	0.78–0.99	0.03 *	0.91	0.80–1.05	0.21
Weight	1	0.93–1.07	0.95			
BMI	1.18	0.92–1.51	0.19			

OR, odds ratio; CI, confidence interval. * significant difference at *p* < 0.05.

**Table 6:** Univariate logistic regression for prediction accuracy of femoral stem size (*n* = 98).

Predictor	Univariate Analysis		
	OR	95% CI	*p*-Value
Female gender	2.04	0.61–6.84	0.25
Height	0.98	0.91–1.06	0.67
Weight	1.00	0.95–1.05	0.97
BMI	1.02	0.89–1.17	0.77

OR, odds ratio; CI, confidence interval.

## Data Availability

The data supporting the findings of this study can be obtained upon request from the corresponding author. The data cannot be made publicly available due to privacy and ethical restrictions.

## Supplementary Materials

The following supporting information can be downloaded at: https://www.mdpi.com/article/10.3390/jcm14248689/s1, Table S1. Mapping table; Table S2. Distribution of implanted component sizes in development and validation cohorts; Table S3. Error distribution (n/N, %) by deviation category for each implant component and templating method; Table S4. Bipolar head prediction accuracy stratified by head size range.

## Abbreviations

The following abbreviations are used in this manuscript:

HA	Hip Arthroplasty
DL	Deep Learning
AP	Anteroposterior
MAE	Mean Absolute Error
RMSE	Root Mean Square Error
AI	Artificial Intelligence
THA	Total Hip Arthroplasty
CT	Computed Tomography
DDH	Developmental Dysplasia of the Hip
BMI	Body Mass Index
CNN	Convolutional Neural Network
LOOCV	Leave-One-Out Cross-Validation
PACS	Picture Archiving and Communication System
ICC	Intraclass correlation coefficient
CI	Confidence Interval
ON	Osteonecrosis
OA	Osteoarthritis
OR	Odd Ratio
ML	Machine Learning
