# Supplementary material for: Development of a Deep Learning Model for Hip Arthroplasty Templating Using Anteroposterior Hip Radiograph
Source: J Clin Med. 2025 Dec 8;14(24):8689. doi: 10.3390/jcm14248689 (PMC12734011; doi:10.3390/jcm14248689)
Supplement: Supplementary file 1 [file jcm-14-08689-s001.zip › jcm-3941172-supplementary.pdf]

Supplements materials

Supplement S1: *The DL model description.*

A grid search was conducted over 45 combinations of 5 learning rates (0.00001, 0.0001,...,0.1), 3 gradient decays (0.9, 0.95, 0.99) , and 3 batch sizes (64, 128, 256). The models were trained using Adam optimizer with fixed epoch setting, and the best configuration was selected based on the lowest validation loss and RMSE.

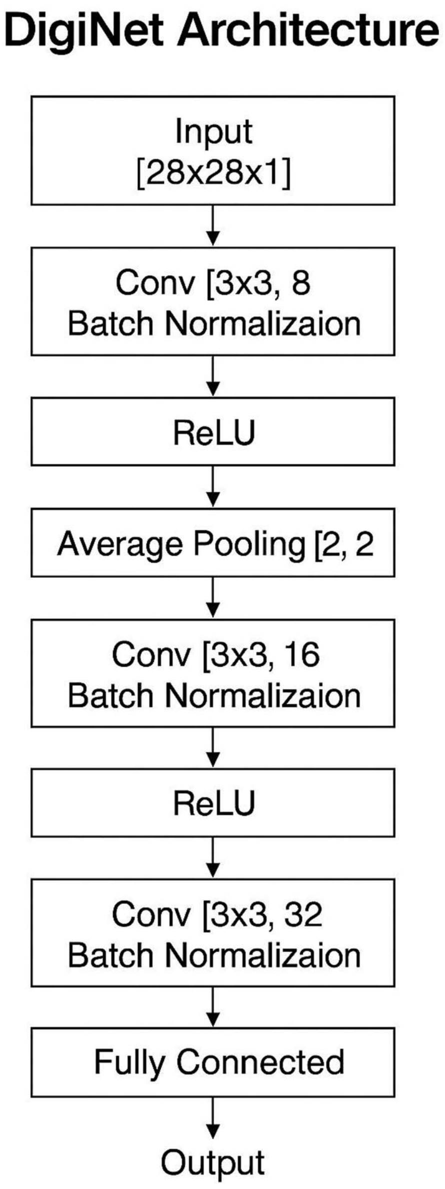

Supplement Table S1:

Supplementary Table S1. Mapping table

Components	Number of sizes	Size range	Increment	Unit
Acetabulum cup	12	44-66	2	mm.
Bipolar head	16	39-54	1	mm.
Femoral stem	11	6,8-18	1 catalog step	step

Supplementary Table S2. Distribution of implanted component sizes in development and validation cohorts.

Component	Size	Development cohort	Validation cohort
Acetabulum	44	6 (1.6%)	-
	46	10 (2.7%)	3 (5.6%)
	48	130 (35.7%)	21 (38.9%)
	50	100 (27.5%)	16 (29.6%)
	52	66 (18.1%)	5 (9.3%)
	54	31 (8.5%)	6 (11.1%)
	56	16 (4.4%)	1 (1.9%)
	58	2 (0.5%)	-
	60	3 (0.8%)	2 (3.7%)
Bipolar head	39	10 (3.1%)	2 (4.5%)
	40	15 (4.7%)	1 (2.3%)
	41	21 (6.5%)	3 (6.8%)
	42	44 (13.7%)	4 (9.1%)
	43	45 (14.0%)	10 (22.7%)
	44	47 (14.6%)	2 (4.5%)
	45	35 (10.9%)	11 (25.0%)
	46	29 (9.0%)	-
	47	21 (6.5%)	1 (2.3%)
	48	17 (5.3%)	-
	49	8 (2.5%)	5 (11.4%)
	50	10 (3.1%)	2 (4.5%)
	51	4 (1.2%)	1 (2.3%)
	52	7 (2.2%)	-
	53	2 (0.6%)	-
	54	6 (1.9%)	-
Femoral stem	6	4 (0.5%)	-
	8	112 (16.3%)	23 (23.5%)
	9	96 (14.0%)	20 (20.4%)
	10	247 (36.0%)	26 (26.5%)
	11	49 (21.7%)	21 (21.4%)
	12	50 (7.3%)	3 (3.1%)
	13	12 (1.7%)	3 (3.1%)
	14	9 (1.3%)	2 (2.0%)
	15	7 (1.0%)	-
	16	1 (0.1%)	-

Supplementary Table S3. Error distribution (n/N, %) by deviation category for each implant component and templating method

Error (Predict–Actual)	Component					
	**Acetabulum (N=54)**		**Bipolar head (N=44)**		**Femoral stem (N=98)**	
	**DL model**	**On-screen**	**DL model**	**On-screen**	**DL model**	**On-screen**
-5	2/54 (3.7 %)	-	-	-	-	-
-4	-	-	-	-	-	1/98 (1.0 %)
-3	-	-	-	-	2/98 (2.0 %)	-
-2	3/54 (5.6%)	2/54 (3.7%)	5/44 (11.4%)	0/44 (0.0%)	6/98 (6.1%)	3/98 (3.1%)
-1	10/54 (18.5%)	7/54 (13.0%)	9/44 (20.5%)	6/44 (13.6%)	21/98 (21.4%)	16/98 (16.3%)
0	24/54 (44.4%)	23/54 (42.6%)	19/44 (43.2%)	27/44 (61.4%)	33/98 (33.7%)	40/98 (40.8%)
+1	14/54 (25.9%)	15/54 (27.8%)	4/44 (9.1 %)	8/44 (18.2%)	30/98 (30.6%)	24/98 (24.5%)
+2	1/54 (1.9%)	5/54 (9.3%)	5/44 (11.4 %)	3/44 (6.8%)	6/98 (6.1%)	12/98 (12.2%)
+3	-	2/54 (3.7 %)	-	-	-	2/98 (2.0 %)
+4	-	-	1/44 (2.3 %)	-		
+5	-	-	1/44 (2.3 %)	-		

Supplementary Table S4. Bipolar head prediction accuracy stratified by head size range.

Head size range	Correct (n)	Total (n)	Accuracy
≤ 42	7	10	70%
43–45	18	23	78.3%
≥ 46	7	11	63.6%
**Total**	**32**	**44**	**72.7%**
